# Frequent Sips of the Water for the Management of Gastroesophageal Reflux Induced Refractory Cough: A Case Report and Review of the Literature

**DOI:** 10.1155/2019/9205259

**Published:** 2019-06-03

**Authors:** Hassan Tariq, Jasbir Makker, Rafeeq Ahmed, Trupti Vakde, Harish Patel

**Affiliations:** ^1^Department of Medicine, BronxCare Hospital System, Bronx, New York 10457, USA; ^2^Division of Gastroenteorlogy, BronxCare Hospital System, Bronx, New York 10457, USA; ^3^Division of Pulmonary and Critical Care Medicine, BronxCare Hospital System, Bronx, New York 10457, USA

## Abstract

**Background:**

Chronic cough is often associated with gastroesophageal reflux disease (GERD). The role of gastroenterologist in the management of the chronic cough is to identify and manage GERD. Ineffective esophageal motility is often associated with GERD induced cough. Chronic cough is often refractory to medical and surgical management despite adequate acid control. Unresponsiveness warrants a thorough pulmonary evaluation. The pathophysiology of refractory cough in these patients is poorly understood, and hence management is often challenging.

**Case Presentation:**

A 75-year-old woman from Ghana was evaluated for GERD associated chronic cough. A 48-hour ambulatory pH study revealed acid exposure of 4.9% and high-resolution manometry showed decreased lower esophageal sphincter pressure, an inadequate response to medical and surgical management of GERD. Postfundoplication ambulatory pH testing demonstrated well-controlled acid reflux but her cough still persisted. Repeat manometry showed an ineffective motility disorder (IEM). Taking frequent sips of water eventually resolved her chronic cough.

**Conclusion:**

Frequent sips of water can be used in the management of the gastroesophageal reflux and ineffective motility induced cough. It results in increased esophageal clearance of acid, nonacid reflux, and ingested pharyngeal secretions, thus breaking the cycle of cough generated increased intra-abdominal pressure with reflux and more cough.

## 1. Introduction

Cough is a protective mechanism to prevent efflux of foreign particulate in the airway. Cough for a duration of more than eight weeks, though debatable, in a nonsmoker and an immunocompetent host is defined as “chronic cough” [1, 2]. It is one of the most common symptoms requiring physician evaluation [3, 4]. Given the excessive use of the respiratory muscles, interference with communication, and sleep deprivation from nocturnal symptoms, chronic cough impacts the quality of the life [5]. The common etiologies of chronic cough are asthma, postnasal drip, and gastroesophageal reflux disease (GERD). In the majority of cases, cough evaluation concludes to inciting factors [2]. Once an etiology is identified, management of the underlying cause results in resolution of chronic cough in majority of patients [6].

Upon consultation, gastroenterologist plays a vital role in establishing an association between chronic cough and GERD. GERD is the second most common etiology for chronic cough [5]. In the absence of typical GERD symptoms, like heartburn, cough can be a sole presentation for this entity. The empiric therapy with two weeks of proton pump inhibitor (PPI) has both a curative and diagnostic intent [7]. Cough resolution is expected in 4 weeks in up to 80% of patients; and in nonresponders esophageal pH monitoring is recommended [8]. The proton pump inhibitor (PPI) unresponsive GERD induced cough can be managed with prokinetic agents. Subsequently, if there is no response to the aforementioned therapy, fundoplication can be considered. Antireflux surgery has revealed promising results for GERD induced cough in patients with elevated acid exposure as studied on the ambulatory pH [8].

Ineffective esophageal motility (IEM) is a distal esophageal motility disorder. On high-resolution manometry (HRM) it is defined as the distal contractile integral (DCI) <100 mmHg/s/cm (failed contraction) or DCI >100 but less than 450 mmHg/s/cm (weak contraction) in 50% or more test swallows [9]. The etiology of IEM is not well understood. In patients with the IEM, there is an increased distal esophageal acid clearance time [10] hence leading to symptoms of GERD. IEM is one of the common esophageal motility abnormalities in patients with GERD, though this association with GERD as defined on ambulatory pH monitoring is debatable [11, 12]. IEM is common in patients with GERD related respiratory symptoms, with cough being the predominant presentation [13].

We present a case of GERD induced chronic refractory cough. GERD diagnosis as confirmed by 48-hour ambulatory pH monitoring required fundoplication for its management. After fundoplication, cough remained refractory despite good acid control as demonstrated on the repeat ambulatory pH recording. The esophageal manometry after fundoplication revealed ineffective motility disorder (IEM). To the best of our knowledge, there is scarcity of literature to guide the management of GERD associated refractory cough after the fundoplication. Our case remains novel, demonstrating that a simple remedy like taking frequent sips of water can result in resolution of GERD associated refractory cough which had persisted despite fundoplication with good acid control.

## 2. Case Presentation

A 75-year-old woman from Ghana with medical comorbidities of hypertension (not on an ACE inhibitor) and chronic cough was referred to our gastroenterology (GI) clinic for management of suspected gastroesophageal reflux disease (GERD) as the cause of chronic cough. As per the patient, she had been having a chronic cough for more than ten years. The cough was nonproductive, without any aggravating or relieving factors. She had reported postprandial heartburn. She recalls that cough started before her heartburn. She reported using albuterol and proton pump inhibitors (PPIs) without improvement in her cough. She did notice some improvement in her heartburn. She had never smoked, and her PPD was negative. There was no prior or current occupational exposure or pet exposure.

She underwent extensive otolaryngology evaluation including a laryngoscopy that showed evidence of chronic laryngopharyngeal reflux. She had been evaluated by pulmonologist and underwent spirometry, imaging studies, bronchoscopy, and fractional exhaled nitric oxide (FENO) testing and all the test results were normal.

She had been prescribed various therapies including oral, nasal, and inhaled corticosteroids, montelukast, and proton pump inhibitors without any improvement in cough.

Because of her typical GERD symptoms, she had a 48-hour Bravo pH testing done in 2011. The study revealed 4.9% of the time with pH below 4 and a total of 106 reflux episodes consistent with GERD. She was evaluated for the surgical intervention, and as a preoperative work-up, high-resolution esophageal manometry was performed. The manometric finding consistent with GERD revealed a hypotensive lower esophageal sphincter (LES). Subsequently, the patient underwent laparoscopic Nissen's fundoplication in 2012.

After fundoplication, she was symptom-free. However, in few months her cough recurred but absence of heartburn and overt acid reflux symptoms was intriguing. She again sought evaluation for cough. She underwent EGD and Bravo pH testing in 2013. There was no evidence of esophagitis and the endoscopic evidence of the fundoplication was appreciated. The ambulatory pH test revealed 0.1% of total acid exposure time and no symptom correlation. She had moved to Ghana for a brief period and did not seek any medical attention. She continued her PPI with no significant improvement.

**Figure 1:**
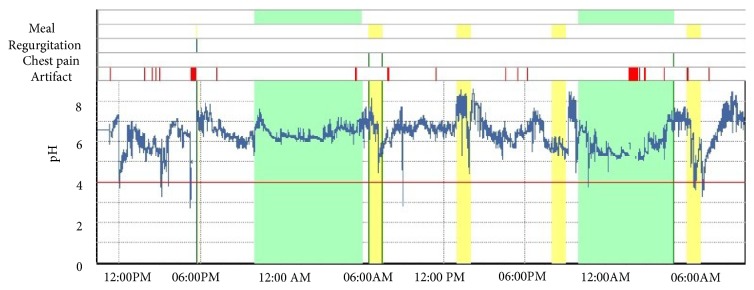
Bravo pH testing did not show any evidence of acid reflux.

Gastroenterology consultation was sought again as the cough persisted. The physical examination including vital signs was unremarkable. She underwent a repeat Bravo pH study in February 2018 that revealed zero acid exposure and the study was not consistent with GERD (Figure 1 and Table 1).

Subsequently, a high-resolution esophageal manometry was done in April 2018 with the findings indicating ineffective esophageal motility (Table 2).

A second opinion was sought from the gastrointestinal motility expert for the medical management of ineffective esophageal motility disorder. Given the absence of dysphagia, she was not considered a good candidate for medical management. To rule out nonacid reflux as a possible etiology of recurrent cough, patient was offered 24-hour Multichannel intraluminal impedance (MII) assisted pH monitoring. However, given the chronic cough and nasal discomfort, patient declined further intervention. Repeat laryngoscopy revealed laryngeal edema. To better assist with esophageal clearance, she was recommended to take frequent sips of water. She was suggested to carry a water bottle and take 1 to 2 sips of water every 15 minutes. Subsequently, patient presented to our clinic for a follow-up visit and was excited to report that her cough after years of work-up and medication use had finally subsided. She reported compliance with frequent sips of water. Patient had multiple interval follow-up for next 6 months where she reported continued absence of cough with multiple sips of water during the day.

## 3. Discussion

Heartburn and acid regurgitation, frequent manifestations of GERD, are the most common symptoms managed by gastroenterologists [14]. Symptom resolution following initial management with proton pump inhibitor (PPI) can conclude the diagnosis [15]. However, it is deemed to have a low specificity of 54% [16].

The extraesophageal manifestations of GERD include asthma, chronic cough, and laryngitis. There is ample evidence to show strong association of GERD with respiratory and laryngeal symptoms [15]. Nevertheless, as suggested in Montreal consensus, it is imperative to conduct thorough evaluation to rule out non-GERD causes for such extraesophageal symptoms [17]. They also opined that extraesophageal symptoms are rare in the absence of typical symptoms of GERD. Individual with chronic cough, even with typical GERD symptoms, should have pulmonary evaluation with imaging studies and bronchoscopy to rule out any pulmonary lesion.

Patients with refractory GERD should have optimization of medical management. For those with refractory symptoms despite optimization, endoscopy and ambulatory pH monitoring is performed for further evaluation. The parameters assessed include presence of hiatal hernia, acid exposure time (AET), and responsiveness to therapy with proton pump inhibitors. Surgical modality can be considered for those with elevated AET (above 6%) and good symptom correlation [18]. However, management is unclear for patients with multiple GERD manifestations like heartburn and cough, where one of the symptoms responds and other does not following therapies targeted against acid control. In our case scenario, heartburn had an appropriate response to PPI, but improvement in cough was minimal. Evaluation for surgical intervention in such a patient needs to be done on a case-to-case basis with a clear understanding of failure to respond with surgical intervention.

It is prudent to understand the advantages as well as shortcomings of different modalities for ambulatory pH testing in the evaluation and management of extraesophageal symptoms like cough. The wireless Bravo® Capsule involves transnasal or peroral route of insertion and suction assisted distal esophageal capsule deployment. The ambulatory catheter-based pH testing includes transnasal pH probe placement with manometry-assisted identification of the lower esophageal sphincter (LES). The catheter is kept for a duration of 24 to 48 hours depending on the protocol [19]. The patient tolerability, multiesophageal site evaluation (distal, proximal, and hypopharyngeal), and the inclusion of the multichannel intraluminal impedance (MII) monitoring are the key features to consider while selecting the modality. The MII-pH system has the multiesophageal sites (distal, proximal, and hypopharyngeal) [20], and the site of the reflux can better assist in understanding the pathogenesis of cough [21]. The impedance monitoring with the MII-pH protocol certainly has advantages of evaluating nonacid reflux; it also assists in assessing correlation of extraesophageal symptoms especially in patients on PPI [22]. In our scenario, the catheter-based MII-pH was not performed; hence, the possibility of the nonacid reflux remains undiagnosed and remains a shortcoming in our case.

Nissen's fundoplication has shown the optimal outcome for gastroesophageal reflux-related heartburn and cough [23]. The postfundoplication recurrence of symptoms requires further evaluation. The fundoplication failure pattern is defined based on the altered surgical anatomy [24]. Essentially, it can lead to the malfunctioning gastroesophageal barrier leading to esophageal acid reflux. The postfundoplication ambulatory pH testing can be performed to assess physiologic characteristics of the gastroesophageal junction [25]. In our case, the optimal acid control was achieved after fundoplication, as evaluated by ambulatory pH monitoring. Postfundoplication persistence of cough with adequate acid control despite resolution of the other symptoms should prompt evaluation for an alternate etiology. A thorough evaluation with the imaging studies, bronchoscopy, and laryngopharyngoscopy was performed in the presented case.

Abnormal esophageal motility has been associated with chronic cough [26]. Gastroesophageal reflux-related respiratory symptoms are also more common in patients with ineffective esophageal motility [13]. It is unclear if the ineffective esophageal motility is the result or the cause of the gastroesophageal reflux [27]. Abnormal motility induced decreased acid clearance can be the likely etiology of chronic cough [13]. The esophageal dysmotility can be a result of fundoplication [28] and requires no further intervention. In our case, cough was optimally controlled for few months after fundoplication. Recurrence of symptoms requires investigation into an alternative explanation. Ineffective esophageal motility resulting from fundoplication could be one of the plausible explanations. It is important to understand the pathophysiology of cough in a patient with gastroesophageal reflux. In refractory cases, evaluation for an underlying etiology of cough is the essence of its treatment.

The prokinetic agents can be utilized in the management of GERD induced cough. The facilitation of the gastric emptying imparts its antitussive action [2]. Their effectiveness in the patients with fundoplication is not clear. Buspirone may also be used for symptomatic management of patients with ineffective esophageal motility. The effectiveness has been demonstrated in management of dysphagia, heartburn, and regurgitation [29]. Buspirone's efficacy, though not proven for cough, can be utilized in a refractory case like ours.

The esophageal-bronchial stimulation can induce GERD related chronic cough [30]. This reflex can be triggered from aspiration related stimulation of upper or lower esophagus without any laryngeal aspiration. Irwin et al. studied the distal and proximal esophageal acid exposure in patients with chronic cough due to GERD and demonstrated that distal esophageal stimulation is the likely etiology of chronic cough [31]. Shaker et al. demonstrated excess of pharyngeal acid exposure in patients with respiratory or laryngeal symptoms [32]. GERD induced reflux laryngitis can also be one of the plausible causes for chronic cough [33]. Aspiration of gastric acid into respiratory tract can be further investigated with bronchoscopy and chest imaging studies [34]. Though not reviewed in literature, patients with fundoplication and esophageal dysmotility may have altered esophageal salivary clearance. The catheter-based MII-pH study along with the laryngoscopy findings can assist in evaluating the direction of the refluxed material and further differentiating the etiology. Taking frequent sips of the water can increase the esophageal clearance [35, 36] and hence can be a remedy to cough by preventing the esophageal-bronchial stimulation.

In our presented scenario, cough may have resulted either from nonacid gastroesophageal reflux or esophageal ineffective motility induced altered pharyngeal secretion clearance. The pathophysiological basis for sips of water resulting in resolution of refractory cough could be the following:

- The sips of the water create the foreign body sensation and inhibit the cough reflex [37].
- It breaks the paroxysm of the cough begetting cough and hence decreases gastroesophageal reflux resulting from recurrent cough.
- It increases the clearance of lower esophageal acid and nonacid refluxed contents and hence inhibits the lower esophageal stimulation induced cough.
- The esophageal ineffective motility related decreased clearance of thick viscous salivary and pharyngeal secretions is assisted with sips of water.

## 4. Conclusion

Chronic cough is a common extraesophageal manifestation of GERD, often related to ineffective motility of esophagus. Cough may stay refractory to medical management, despite optimal acid control. The unresponsive cases warrant thorough pulmonary evaluation. Patients with increased acid exposure and symptom correlation can be considered for fundoplication. The refractoriness to medical and surgical management necessitates reevaluation with repeat endoscopy, ambulatory pH testing, and esophageal manometry. The frequent sip of water, a simple remedy, has not been utilized for management of GERD induced cough. The increased clearance of the esophageal secretions as a result of taking frequent sips of water interrupts the vicious cycle of cough and GERD underscores its utility in the management of GERD induced cough.

## Figures and Tables

**Table 1:** Bravo pH parameters and patients study values.

Bravo pH parameters	Patients study values
Acid Exposure Time(s) for pH < 4.0	0

Number of Reflux Episodes	0

Demeester score Total	0.3

**Table 2:** Manometry findings consistent with ineffective esophageal motility.

Manometry parameter	Patients study values
Mean IRP	17 mmHg (Normal < 20)

Mean DCI	459mmHg.sec.cm (Normal <5000)

Normal DCI (450 - 8000)	38.5 %

Mean DL	11 sec (Normal >4.5)

Ineffective	61.5 % (Normal < 50.0%)

Hypercontractile (DCI 450 – 8000)	0 % (Normal <20.0%)

Premature (DL < 4.5)	0 % (Normal <20.0%)

Pattern Classification	Ineffective esophageal motility

Transit Completed	30.8 % swallows

Distal esophageal amplitude	33 mmHg (Normal <= 220)
